# Impact of gram negative bacteria airway recolonization on the occurrence of chronic lung allograft dysfunction after lung transplantation in a population of cystic fibrosis patients

**DOI:** 10.1186/s12866-018-1231-7

**Published:** 2018-08-20

**Authors:** Sarah Orfanos, Carine Gomez, Sophie Baron, Ritesh Akkisetty, Nadine Dufeu, Bérengère Coltey, Pascal Alexandre Thomas, Jean Marc Rolain, Martine Reynaud-Gaubert

**Affiliations:** 1 Aix-Marseille University, Faculté de médecine, Marseille, France; 2 Department of Respiratory Diseases, Lung Transplant Team, University Hospital of Marseille, Marseille, France; 3 URMITE CNRS IRD UMR 6236, IHU Méditerranée Infection, Aix-Marseille University, Marseille, France; 4 Department of Biology, Drexel University College of Art and Sciences, Philadelphia, USA; 5 Department of Thoracic Surgery, Lung Transplant Team APHM, University Hospital of Marseille, Marseille, France

**Keywords:** Chronic lung allograft dysfunction, Lung transplantation, Infection and inflammation, Cystic fibrosis

## Abstract

**Background:**

Chronic Lung Allograft Dysfunction (CLAD) is the main cause of morbidity and mortality after the first year following lung transplantation (LTx). Risk factors of CLAD have been extensively studied, but the association between gram-negative bacteria (GNB) bronchial colonization and the development of CLAD is controversial. The purpose of our study was to investigate the association between post-transplant recolonization with the same species or de-novo colonization with a new GNB species and CLAD. The same analysis was performed on a sub-group of patients at the strain level using Matrix Assisted Laser Desorption Ionization-Time of Flight Mass Spectrometry technique.

**Results:**

Forty adult cystic fibrosis (CF) patients who underwent a first bilateral LTx in the University Hospital of Marseille, between January 2010 and December 2014, were included in the study. Patients with GNB de-novo colonization had a higher risk of developing CLAD (OR = 6.72, *p* = 0.04) and a lower rate of CLAD-free survival (*p* = 0.005) compared to patients with GNB recolonization. No conclusion could be drawn from the subgroup MALDI-TOF MS analysis at the strain level.

**Conclusion:**

Post-LTx GNB airway recolonization seems to be a protective factor against CLAD, whereas de-novo colonization with a new species of GNB seems to be a risk factor for CLAD.

**Electronic supplementary material:**

The online version of this article (10.1186/s12866-018-1231-7) contains supplementary material, which is available to authorized users.

## Background

Chronic lung allograft dysfunction (CLAD) is the major factor limiting long term survival following lung transplantation (LTx). The International Society of Heart and Lung Transplantation (ISHLT) evaluates the prevalence of bronchiolitis obliterans syndrome (BOS) at 50% at 5 years post transplantation [1].

Previously known as “chronic rejection”, the term CLAD was coined in 2010 in order to encompass two distinct identities: BOS and restrictive allograft syndrome (RAS) [2, 3]. The underlying physiopathological mechanisms leading to the development of CLAD are yet to be fully understood [4]. However, several risk factors have been identified that have a significant association with the development of BOS, among which: primary graft dysfunction, acute cellular or humoral rejection, lymphocytic bronchiolitis, gastro-esophageal reflux, viral, fungal or bacterial pneumonitis or bronchial infection [5]. Of these risk factors, bronchial infection remains controversial. While some describe it as significantly associated with the development of BOS [6–9]; others have refuted this association [10, 11]. Rare are the longitudinal studies concluding if post-transplant bronchial colonization with the same species of gram negative bacteria (GNB) impacts allograft functional outcome [11].

In order to study bacterial populations, the use of Matrix Assisted Laser Desorption Ionization-Time of Flight Mass Spectrometry (MALDI-TOF MS) has shown its efficacy in the field of proteomics for the identification of routinely isolated micro-organisms when compared to conventional phenotypic identification [12]. It was also demonstrated that MALDI-TOF MS is efficient in the identification of difficult to identify bacterial strains [13], anaerobes [14], and in patients with multiple bacterial colonizations as observed in cystic fibrosis (CF) patients [15, 16]. Unlike conventional phenotypic identification, MALDI-TOF MS enables strain level typing within the same bacterial species. It is considered as sensitive and accurate as the gold standard (16S rRNA PCR) while being less expensive than molecular biology [17–20].

The objective of our study was to evaluate the impact of Gram Negative Bacteria (GNB) colonization of the lung allograft on the development of CLAD, in a population of patients with CF. Patients with GNB de-novo colonization are hypothesized to have a higher risk of CLAD development compared to patients without GNB de-novo colonization.

## Methods

### Study population

A retrospective observational single-center study was conducted on a population of adult CF patients who underwent a first LTx or heart-LTx at the University Hospital of Marseille, France, between January 2010 and December 2014. Patients who were included in the analysis had at least two culture samples (bacterial sputum culture (BSC), bronchial aspirates (BA) or broncho-alveolar lavage (BAL)) during the postoperative period (at 1, 6 or 12 months post-LTx).

For the subgroup analysis using MALDI-TOF MS technique, patients who did not have at least one specimen available both pre and post-LTx for the same bacterial species were excluded.

Currently, there is no consensus regarding the definition of chronic pulmonary colonization [21]. In this study, colonization was defined as the isolation of the same bacterial species at least twice consecutively at three weeks interval. Patients for whom only one culture was available, infection was ruled out by reviewing blood count, chest X-ray and whether this was a routine sample collection or triggered by clinical symptoms. Recolonization at species level was defined as the isolation of the same bacterial species pre and post-LTx. Strain level re-colonization was defined by the identification of the same bacterial strain through MALDI- TOF MS analysis pre-LTx and post-LTx. De-novo colonization was defined by the identification of a new bacterial species (culture) or bacterial strain (MALDI-TOF MS) post-LTx.

We also took into account risk factors recognized as associated with CLAD [5, 22, 23]: CMV active replication, filamentous fungal colonization in the 6 months preceding LTx and in the first year post-LTx, histologically proven acute cellular rejection (grade > A2) [24] with trans-bronchial biopsies done following international guidelines [25].

Written informed consent was obtained from all patients. The study was approved by the local ethic committee (Assistance Publique Hôpitaux de Marseille).

### Immunosuppressive regimen and anti-infectious prophylaxis protocols

All recipients received a standardized immunosuppressive regimen. Induction therapy consisted of intravenous administration of 1.5 mg/kg/day of rabbit anti-thymocyte globulins given for the first three postoperative days, associated with a high dose of methylprednisolone. Intravenous cyclosporine was administered immediately after LTx and was then switched to oral tacrolimus. Standard triple maintenance immunosuppressive regimen consisted of tacrolimus, mycophenolate mofetil and prednisone.

Postoperatively, recipients received a prophylactic antibiotic treatment according to their preoperative and/or concomitant infectious status, for at least 14 days. Seropositive CMV recipients received prophylactic IV ganciclovir for the first two postoperative weeks. Higher risk CMV-mismatched recipients (Donor+/Recipient-) were treated systematically for the first 3 months. Antifungal prophylaxis with voriconazole was used during the first month in case of previous fungal infection present in the 6 months preceding LTx. Our center did not practice routine prophylaxis against *Pneumocystis* pneumonia.

### Pulmonary function tests (PFTs) and CLAD definition

PFTs were performed according to the American Thoracic Society and European Respiratory Society guidelines [26]. Lung function monitoring was done by extensive PFTs, weekly during the first month, monthly in the first year post-transplant and when clinically indicated.

The diagnosis of CLAD was made on the PFTs according to the ISHLT’s definition [3, 5]. BOS was defined as a decline of ≥20% in the forced expiratory volume in 1 s (FEV1), compared to the mean of the two best successive post-operative values, obtained at three weeks interval, without other identifiable causes [5]. RAS was defined as a decline of ≥10% in total lung capacity (TLC) in addition to an interstitial pattern on chest tomography [27, 28].

### Sample acquisition and analysis

Sputum samples were collected when clinically indicated. BAL and BA were obtained during a bronchoscopy, following current guidelines [25]. Samples were cultured on 4 different media: MacConkey agar (Biomérieux, Marcy l’Etoile, France), chocolate agar (Biomérieux), 5% sheep blood enriched Columbia agar with nalidix acid and colistin (ANC Columbia) (Biomérieux) and Cepacia agar (BD, Franklin Lakes, New Jersey, United States).

Bacterial colonies were identified using MALDI-TOF MS as previously described [29]. Antimicrobial susceptibility testing was performed according the European Committee on Antimicrobial Susceptibility Testing (EUCAST) recommendation. MALDI-TOF was performed on all samples at the time of collection. Bacterial spectra obtained by MALDI- TOF MS for each strain were recovered, classified by species and used to build specific dendrogram (Bruker Biotyper 3 software) for each species. Strains were considered identical if the distance separating them on the dendrogram was less than 500.

### Statistical analysis

Categorical data were expressed in percentages and absolute values. Continuous data were expressed in means and standard deviations. Categorical data were compared using Fisher’s Exact test in the univariate analysis. The multivariate analysis was done using a stepwise, multivariate, logistic regression model. Continuous data were compared using Mann-Whitney nonparametric tests. Survival analysis were performed using the Kaplan-Meier estimate and compared using the log rank test. Two sided *P* values < 0.05 were considered statistically significant.

## Results

### Study population

During the study period, fifty-five CF adult patients underwent LTx in our transplant center. Of these patients, forty CF lung transplant recipients had at least two bronchial cultures available in the 6 months pre-transplant and at 1, 6 and/or 12 months post-transplant and were included in the study. All the patients included in the study were followed for more than a year, 34 patients for more than 2 years, 26 patients for more than 3 years and 21 patients for more than 4 years. A mean follow-up of 1413.5 days (±565.8) was reported. Baseline characteristics are reported in Table 1. A total of 9 patients developed CLAD over the study period (7 BOS and 2 RAS). The total incidence rate of CLAD in our population was 67 cases per 1000 person-years.

**Table 1:** Patient demographics (*n* = 40)

	Total n: 40	GNB recolonization n: 28	GNB de-novo colonization n: 7	Exempt of GNB n: 5	*p*
Age, years	28.5 ± 10.46	28.6 ± 9.2	25.4 ± 8.9	34.2 ± 15	0.33
Sex, male	19 (47.5%)	14 (50%)	4 (57%)	1 (20%)	0.40
High emergency LTx	7 (17.5%)	6 (21.4%)	0	1 (20%)	0.40
Bilateral LTx	39 (97.5%)	28 (100%)	6 (85.7%)	5 (100%)	0.09
HLTx	1 (2.5%)	0	0	0	0.09
Lung ischemic time, minutes	355 ± 86.1	360 ± 99.5	351 ± 41.1	330 ± 33.2	0.77
CMV D+/R-	4 (10%)	2 (7.1%)	1 (14.3%)	1 (20%)	0.62
ICU length of stay, days	25.2 ± 33.2	30.8 ± 38	8.4 ± 5.2	17.2 ± 12	0.24
Length of invasive mechanical ventilation, days	17.5 ± 32.4	21.9 ± 38.3	3.7 ± 3.3	6.2 ± 5.5	0.36
Follow up, days	1413.5 ± 565.8	1509.3 ± 527.8	1044.3 ± 590.7	1394.2 ± 643.2	0.15
One year survival, %	100	100	100	100	
Best post-transplant FEV1, %predicted	83.8 ± 18	82.5 ± 19.3	90.8 ± 12.6	82.2 ± 16.7	0.59
CLAD	9 (22.5%)	3 (10.7%)	4 (57%)*	2 (40%)	0.02*
BOS	7 (17.5%)	3 (10.7%)	2 (28.6%)	2 (40%)	0.20
Time to BOS, months	36.4 (20.2)	27.3 (18.5)	25.5 (6.4)	61 (5.7)	0.09
RAS	2 (5%)	0	2 (28.6%)*	0	0.007*
Time to RAS, months	13	NA	13	NA	

Definition of abbreviations: *GNB* Gram Negative Bacteria, *LT* Lung Transplant, *HLT* Heart Lung Transplant, *ICU* Intensive Care Unit, *FEV1* Forced Expiratory Volume in 1 s, *CLAD* Chronic Lung Allograft Dysfunction, *BOS* Bronchiolitis Obliterans Syndrome, *RAS* Restrictive Allograft Syndrome. Data are expressed in n (%) and means±SDs

### Bacterial epidemiology

Bacterial airways’ colonization results are reported in Table 2 (Additional file 1). During the six pre-operative months, a sample of GNB was isolated in all forty transplanted patients, 90% of whom (*n* = 36) were colonized with *Pseudomonas aeruginosa (PA)*. At one month, six months and twelve months post-LTx respectively 57.5% (*n* = 23), 64.5% (*n* = 20) and 51.5% (*n* = 17) were colonized with a GNB.

**Table 2:** Pre and post- lung transplant colonizations

	Pre transplant (*n* = 40)	Month 1 (*n* = 40)	Month 6 (*n* = 31)	Month 12 (*n* = 33)
GNB	40 (100)	23 (57.5)	20 (64.5)	17 (51.5)
Pseudomonas aeruginosa	36 (90)	21 (52.5)	16 (51.6)	16 (48.5)
Recolonization	N/A	20 (95.2)	15 (93.7)	15 (93.7)
De-novo	N/A	1 (4.8)	1 (6.3)	1 (6.3)
Achromobacter xylosoxidans	4 (10)	2 (5)	2 (6.4)	0
Recolonization	N/A	0	1 (50)	N/A
De-novo	N/A	2 (100)	1 (50)	N/A
Stenotrophomonas maltophilia	4 (10)	1 (2.5)	0	0
Recolonization	N/A	0	N/A	N/A
De-novo	N/A	1 (100)	N/A	N/A
Alcaligenes xylosoxidans	3 (7.5)	2 (5)	0	0
Recolonization	N/A	2 (100)	N/A	N/A
De-novo	N/A	0	N/A	N/A
Pandorea pulmonicola	2 (5)	2 (5)	2 (6.4)	1 (3)
Recolonization	N/A	2 (100)	2 (100)	1 (100)
De-novo	N/A	0	0	0
Proteus mirabilis	1 (2.5)	1 (2.5)	0	1 (3)
Recolonization	N/A	1 (100)	N/A	0
De-novo	N/A	0	N/A	1 (100)
Serratia marcescens	1 (2.5)	1 (2.5)	0	0
Recolonization	N/A	1 (100)	N/A	N/A
De-novo	N/A	0	N/A	N/A
Burkholderia multivorans	1 (2.5)	1 (2.5)	1 (3.2)	1 (3)
Recolonization	N/A	1 (100)	1 (100)	1 (100)
De-novo	N/A	0	0	0
Acinetobacter baumanii	1 (2.5)	0	0	0
Recolonization	N/A	N/A	N/A	N/A
De-novo	N/A	N/A	N/A	N/A
Klebsiellae pneumoniae	0	1 (2.5)	0	0
Recolonization	N/A	1 (100)	N/A	N/A
De-novo	N/A	0	N/A	N/A
Moraxella catarrhalis	0	0	1 (3.2)	0
Recolonization	N/A	N/A	0	N/A
De-novo	N/A	N/A	1 (100)	N/A
Haemophilus influenzae	0	0	1 (3.2)	0
Recolonization	N/A	N/A	0	N/A
De-novo	N/A	N/A	1 (100)	N/A
Escherichia coli	0	0	1 (3.2)	0
Recolonization	N/A	N/A	0	N/A
De-novo	N/A	N/A	1 (100)	N/A
Mycobacterium abscessus	1 (2.5)	0	0	0
Recolonization	N/A	N/A	N/A	N/A
De-novo	N/A	N/A	N/A	N/A
Staphylococcus aureus	12 (30)	6 (15)	10 (32.3)	8 (24.2)
Recolonization	N/A	3 (50)	4 (40)	2 (25)
De-novo	N/A	3 (50)	6 (60)	6 (75)

Definition of abbreviations: *GNB* Gram Negative Bacteria. Data are expressed in n (%)

### MALDI-TOF MS analysis

Of the forty patients included in the study, fourteen patients had at least one MALDI-TOF MS spectrum available pre and post-LTx, enabling the construction of dendrograms (Additional file 2). For thirteen patients, the comparison of *PA* strains from these two periods showed that seven patients were colonized with the same *PA* strain, two patients were both recolonized and de- novo colonized and four patients had a new strain of *PA*. The two patients colonized with *Pandorea pulmonicola (PP)* in the study, were colonized with the same strain of *PP* pre and post-LTx.

### Effect of GNB recolonization and de-novo colonization of the lung allograft on the development of CLAD, overall survival and CLAD-free survival

The incidence of each risk factor known to be associated with the development of CLAD is reported in Table 3. None of these risk factors were significantly associated with the development of CLAD in our study (Table 4).

**Table 3:** Incidence of chronic lung allograft dysfunction risk factors in our population (*n* = 40)

Risk factor	n (%)
Acute rejection	23 (57.5%)
Post-LTx CMV replication	7 (17.5%)
Post-LTx filamentous fungi colonization	17 (42.5%)
Post-LTx Aspergillus colonization	12 (30%)

Definition of abbreviations: *LTx* Lung Transplant, *CMV* cytomegalovirus

**Table 4:** Association between different risk factors and CLAD (univariate and multivariate analysis)

	OR (univariate)	*P* value	OR (multivariate)	*P* value
Acute rejection	1.65	0.71	1.21	0.83
Post-LT CMV replication	0.52	1	0.51	0.61
Post-LT filamentous fungi colonization	1.73	0.70	1.27	0.78
GNB de-novo colonization	11.11	0.018*	6.72	0.04*

Definition of abbreviations: *LT* Lung Transplant, *CMV* Cytomegalovirus, *GNB* Gram negative Bacteria, *OR* Odd Ratio, *: *p*<0.05

**Fig. 1:**
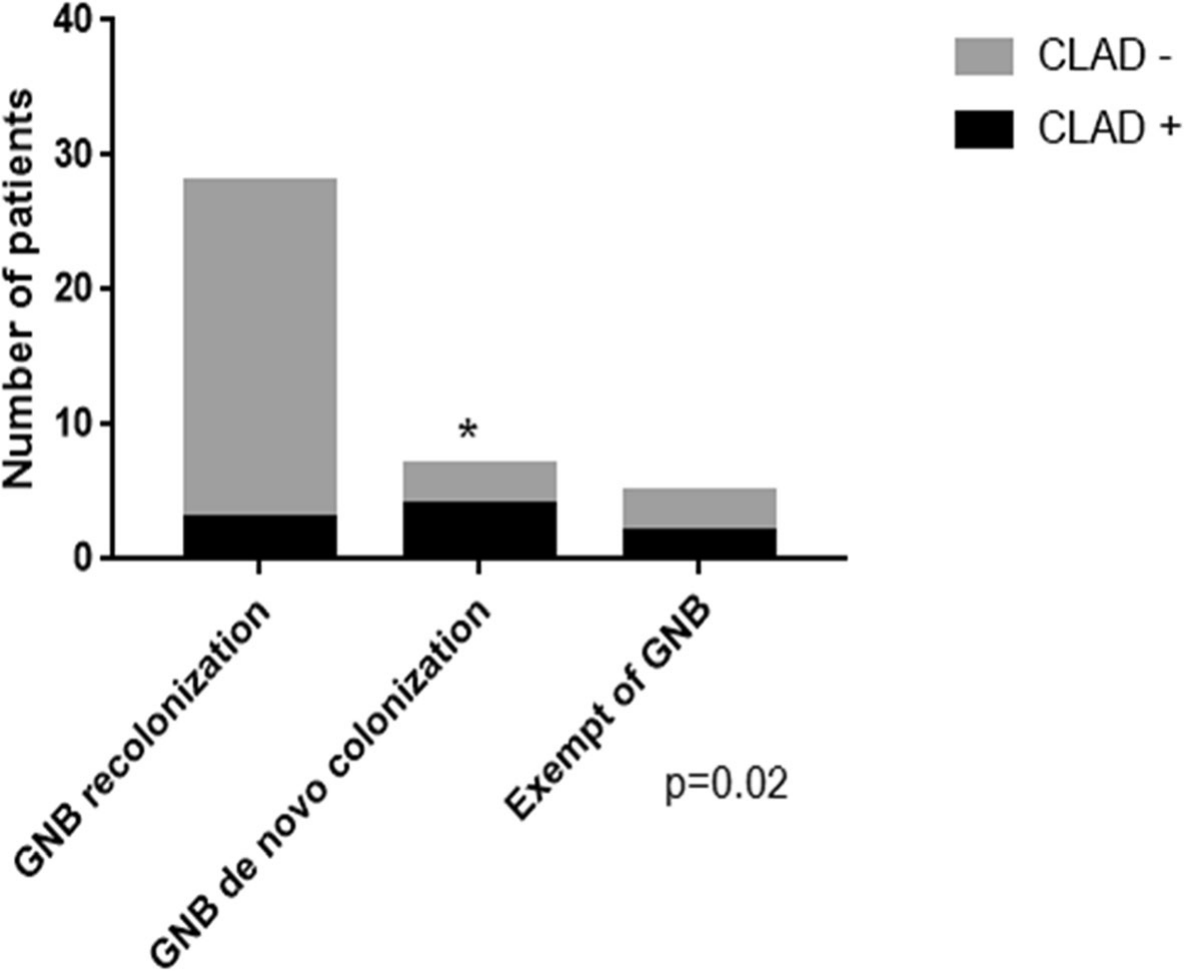
Number of chronic lung allograft dysfunction in the three groups: GNB recolonization, GNB de novo colonization and exempt of GNB. Definition of abbreviations: CLAD: Chronic Lung Allograft Dysfunction, GNB: Gram Negative Bacteria. The symbol * indicates statistical significance. The group colonized with a new species of GNB post lung transplant exhibited a higher incidence of chronic lung allograft dysfunction compared to the patients recolonized with the same species of GNB or the patients free from GNB colonization (*p* = 0.02)

The forty patients who met the aforementioned inclusion criteria were categorized into three exclusive groups: GNB recolonization without de-novo colonization *n* = 28 (70%), de-novo colonization *n* = 7 (17.5%) and no measurable GNB colonization *n* = 5 (12.5%). The incidence of CLAD was highest in the group with de-novo colonization (*p* = 0.02) (Fig. 1). Patients with de-novo colonization had a higher risk of developing CLAD than patients who were recolonized with the same species of GNB, in univariate analysis (OR = 11.11, *p* = 0.018, [1.63, 75.60]), and multivariate analysis (OR = 6.72, *p* = 0.04, [1.04, 43.24]) (Table 4).

**Fig. 2:**
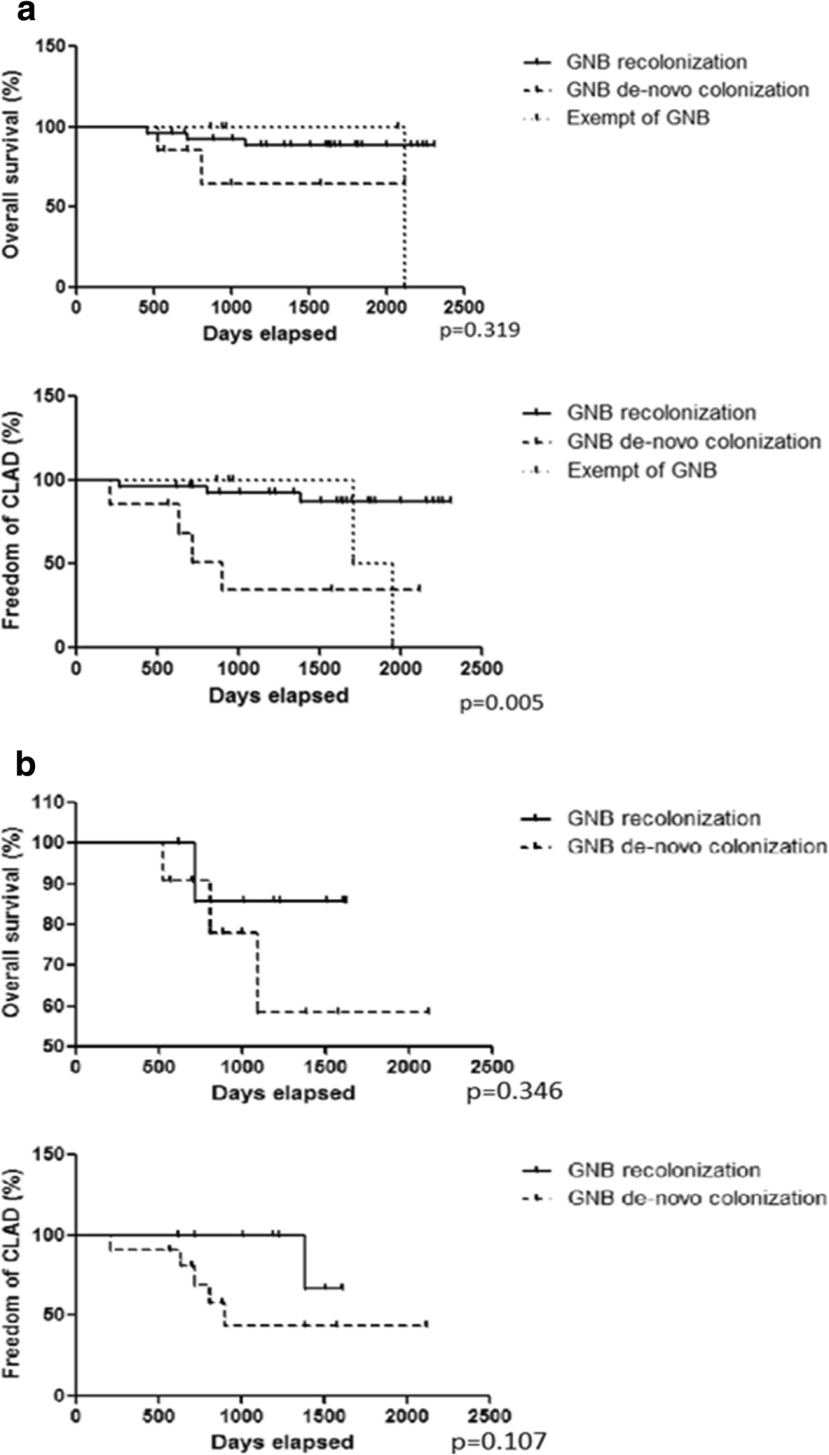
Overall and CLAD-free survival in the 3 groups: GNB recolonization, GNB de novo colonization and exempt of GNB: **a** At the species level (conventional phenotypic identification). **b** At the strain level (MALDI-TOF MS). Definition of abbreviations: CLAD: Chronic Lung Allograft Dysfunction, GNB: Gram Negative Bacteria. At the species level the group: GNB de-novo colonization presented with a worse CLAD free survival than the groups GNB recolonization or exempt of GNB colonization (*p* = 0.005). At the strain level there was a tendency to better CLAD free survival in the subgroup GNB recolonization compared to the subgroup de-novo GNB colonization without reaching statistical significance

The rate of CLAD free survival was lower in the patients with de-novo colonization than in the patients presenting re-colonization (*p* = 0.005). There was no significant difference in overall survival between the 3 groups (*p* = 0.319) (Fig. 2).

A subgroup analysis was performed for patients for whom MALDI-TOF MS spectrum pre- and post-LTx were available (*n* = 14). There were no significant differences in overall survival (*p* = 0.346) and CLAD free survival (*p* = 0.107) between the three groups (Fig. 2).

There were no significant differences in the best post-LTx FEV1 between the three groups (Additional file 3).

## Discussion

Few studies have performed a longitudinal analysis of airway bacterial colonization pre-LTx and post-LTx in a population of CF patients. We used proteomics to complement conventional phenotypic identification of micro-organisms. Comparison of MALDI-TOF MS spectrum enabled us to determine if post-LTx colonization was due to the same strain. Our study has shown in concordance with the previous study by Willner et al. [11], that post-LTx colonization with a new GNB is a risk factor to develop CLAD and worsens CLAD-free survival, whereas recolonization with the same GNB is a protective factor against the development of CLAD and improves CLAD-free survival.

This study has enabled a thorough and exhaustive record of pre and post-LTx airways’ colonization in the CF population of our center. The bacterial epidemiology reported in the study’s population was comparable to other studies [7, 8, 11, 30, 31], as well as the rate of GNB recolonization (70%) and the rate of GNB de novo colonization (17.5%) following LTx [8].

For fourteen of our patients, we were able to compare pre and post-LTx MALDI-TOF MS spectrum. This approach, unlike conventional bacterial culture, enabled strain level typing within a species with accuracy while being more cost and time efficient than molecular biology [13]. Assuming the existence of an extensive data base [32], the proteomic approach can be considered as sensitive as the GOLD standard (16S rRNA PCR) [16]. In accordance with previous study, the construction of dendrograms for recolonizing species has shown that almost 70% of patients were recolonized with the same strain of *PA* they exhibited prior to LTx [33, 34]. In the subgroup of patients who had accessible MALDI-TOF spectrum, groups were readjusted for re-colonization and de-novo colonization at the strain level, but the statistical power of the analysis in this subgroup was too low to confirm a difference in CLAD-free survival between the two groups, although there was a tendency to better CLAD free survival in the recolonized group.

The association between BOS and GNB post-transplant colonization has been debated in recent literature [6–11]. In this study, we have taken into account possible confounding factors considered as risk factors for CLAD (acute rejection, CMV replication, filamentous fungi colonization) [5, 9, 22, 35], however, none of them were associated with CLAD in our univariate and multivariate analysis. Unfortunately due to incomplete medical records we did not include primary graft dysfunction, a well-known risk factor for BOS, in our multivariate analysis. Moreover with the knowledge that CLAD is a time dependent phenomenon, a logistic regression integrating follow-up time and CLAD was attempted, unexpectedly patients developing CLAD had a significantly shorter follow-up (*p* = 0.02) secondary to lower survival. The results of this study concluded that GNB de-novo colonization is a risk factor for the development of CLAD (OR = 6.72, *p* = 0.04). Moreover, GNB re-colonization conveyed an improved CLAD-free survival compared to the group GNB de-novo colonization (*p* = 0.005). These results corroborate findings from Botha et al. and Willner et al. studies, in which an association between GNB de-novo colonization and BOS was found [6, 11]. This study complements Willner et al. study, in which 26 CF patients had samples available pre and post-LTx. With a larger population of 40 CF, our study confirmed that GNB recolonization conveys a protection from CLAD [11]. Therefore the distinction between recolonization and de-novo colonization seems to be of utmost importance when studying the association with CLAD, yet this distinction was overlooked in the first studies published [7]. This would explain why CF lung transplant patients are more frequently colonized with GNB than other lung transplant patients and yet do not exhibit an increase in BOS [31, 36]. The mechanisms linking post-transplant colonization and BOS still need to be fully understood. The dysregulation in the pro and anti-inflammatory cytokines and the increase in polynuclear neutrophils recruitment triggered by *PA* colonization is responsible for chronic airways inflammation, which might lead to the development of BOS [9, 37, 38]. Recently, Bernasconi et al. has demonstrated that the function of the lung allograft was potentially determined by the cross-talk between bacterial communities and innate immune cells [39]. Moreover de-novo colonization has been reported to trigger higher inflammation than recolonization, as chronic colonization of CF patients with *PA* leads to loss of function and reduced virulence [40–42].

Several limitations have been identified in this study. Most of our pre-transplantation samples available were BSC’s (92.5%), the close follow-up required after a lung transplant allowed us to use BAs and BAL for 80% of our patients at one month and 42% of our patients after one month. However the inconsistent use of BSCs, BAs, and BAL to determine colonization in the airways, increases the possibility of contamination as well as decreases sensitivity [43]. Some studies have shown possible discrepancies between lung biopsies and BSCs, but these differences are primarily an issue for satellite bacteria more than for core bacteria. We also chose to study the association between airway colonization and CLAD as a whole. BOS and RAS have different pathogenesis and histologic findings. If some histologic features overlap between the two phenotypes with obliterative bronchiolitis lesions present in both BOS and RAS, RAS is also characterized by the expansion of fibrous connective tissue and the formation of interstitial scar tissue [4]. It is unclear if the inflammation secondary to a new airway colonization can lead to both obliterative bronchiolitis and fibrosis. Therefore the impact of GNB de-novo colonization might have been different if we had studied BOS and RAS separately, although it appears that risk factors for these two phenotypes do not differ [22]. Finally, the subgroup used for the strain level analysis (MALDI-TOF MS) was too small to confirm an association between de-novo colonization and CLAD at the strain level.

## Conclusions

In our study, at the species level, post-transplant GNB de-novo colonization was a risk factor for the development of CLAD and decreased CLAD free survival, compared to patients with post-transplant GNB recolonization. This stresses the importance of GNB species switching and the resulting immune response as a potential target in the prevention of CLAD. A larger longitudinal study with strain level typing either via MALDI-TOF MS or molecular biology is needed to confirm this association.

## Additional files

Additional file 1: Pre and post-lung transplant airway colonization. Pre and post lung transplant airway colonization at the individual level for all forty patients. (DOCX 13 kb)

Additional file 2: *Pseudomonas aeruginosa* dendrogram. MSP dendrogram performed using Biotyper v 3.0, including 52 spectra of *Pseudomonas aeruginosa* isolated before, at one month, six months or twelve months after lung transplant for the 13 patients for which data were available. * indicates isolates of the same patient belonging to the same strain. Each color is specific for one patient. (DOCX 120 kb)

Additional file 3: Best post-lung transplant Forced expiratory volume in 1 s (FEV1) in the three groups: GNB recolonization, GNB de-novo colonization, exempt of GNB. Best post-lung transplant Forced expiratory volume in 1 s (FEV1) in the three groups: GNB recolonization, GNB de-novo colonization, exempt of GNB expressed in percentage of the expected value. (DOCX 13 kb)

## Abbreviations

BA: Bronchial Aspirates
BAL: BronchoAlveolar Lavage
BOS: Bronchiolitis Obliterans Syndrome
BSC: Bacterial Sputum Culture
CF: Cystic Fibrosis
CLAD: Chronic lung allograft dysfunction
CMV: Cytomegalovirus
FEV1: Forced Expiratory Volume in 1 s
GNB: Gram Negative Bacteria
ISHLT: The International Society of Heart and Lung Transplantation
LTx: Lung Transplantation
MALDI-TOF MS: Matrix Assisted Laser Desorption Ionization-Time of Flight Mass Spectrometry
*PA*: *Pseudomonas aeruginosa*
PFT: Pulmonary Function Test
*PP*: *Pandorea pulmonicola*
RAS: Restrictive Allograft Syndrome
TLC: Total Lung Capacity
