# Supplementary material for: Impact of gram negative bacteria airway recolonization on the occurrence of chronic lung allograft dysfunction after lung transplantation in a population of cystic fibrosis patients
Source: BMC Microbiol. 2018 Aug 20;18:88. doi: 10.1186/s12866-018-1231-7 (PMC6102836; doi:10.1186/s12866-018-1231-7)
Supplement: Supplementary file 1 — Pre and post-lung transplant airway colonization. Pre and post lung transplant airway colonization at the individual level for all forty patients. (DOCX 13 kb) [file 12866_2018_1231_MOESM1_ESM.docx]

|  | Pre-transplantation | Post-Transplantation |
| --- | --- | --- |
| Patient 1 | Pseudomonas Aeruginosa, Burkholderia Multivorans, Staphylococcus Aureus | Pseudomonas Aeruginosa, Burkholderia Multivorans, Staphylococcus Aureus |
| Patient 2 | Pseudomonas Aeruginosa | Pseudomonas Aeruginosa |
| Patient 3 | Pseudomonas Aeruginosa, Staphylococcus Aureus | Pseudomonas Aeruginosa, Staphylococcus Aureus |
| Patient 4 | Pseudomonas Aeruginosa, Alcaligenes Xylosoxidans | Pseudomonas Aeruginosa, Alcaligenes Xylosoxidans |
| Patient 5 | Pseudomonas Aeruginosa | Pseudomonas Aeruginosa |
| Patient 6 | Pseudomonas Aeruginosa, Staphylococcus Aureus | Pseudomonas Aeruginosa, Staphylococcus Aureus |
| Patient 7 | Pseudomonas Aeruginosa, Staphylococcus Aureus | Pseudomonas Aeruginosa, Staphylococcus Aureus |
| Patient 8 | Pseudomonas Aeruginosa | Pseudomonas Aeruginosa |
| Patient 9 | Pseudomonas Aeruginosa | Pseudomonas Aeruginosa |
| Patient 10 | Pseudomonas Aeruginosa, Staphylococcus Aureus | Pseudomonas Aeruginosa, Staphylococcus Aureus |
| Patient 11 | Pseudomonas Aeruginosa | Pseudomonas Aeruginosa |
| Patient 12 | Pseudomonas Aeruginosa, Staphylococcus Aureus | Pseudomonas Aeruginosa, Staphylococcus Aureus |
| Patient 13 | Pseudomonas Aeruginosa, Staphylococcus Aureus | Pseudomonas Aeruginosa |
| Patient 14 | Pseudomonas Aeruginosa | Pseudomonas Aeruginosa |
| Patient 15 | Pseudomonas Aeruginosa | Pseudomonas Aeruginosa |
| Patient 16 | Pseudomonas Aeruginosa | Pseudomonas Aeruginosa |
| Patient 17 | Pseudomonas Aeruginosa, Pandorea Pulmonicola | Pseudomonas Aeruginosa, Pandorea Pulmonicola |
| Patient 18 | Pseudomonas Aeruginosa | Pseudomonas Aeruginosa |
| Patient 19 | Pseudomonas Aeruginosa, Staphylococcus Aureus | Pseudomonas Aeruginosa |
| Patient 20 | Pseudomonas Aeruginosa, Staphylococcus Aureus | Pseudomonas Aeruginosa |
| Patient 21 | Pseudomonas Aeruginosa | Pseudomonas Aeruginosa |
| Patient 22 | Pseudomonas Aeruginosa, Staphylococcus Aureus | Pseudomonas Aeruginosa, Staphylococcus Aureus |
| Patient 23 | Pseudomonas Aeruginosa | Pseudomonas Aeruginosa |
| Patient 24 | Pseudomonas Aeruginosa, Staphylococcus Aureus, Stenotrophomonas Maltophilia | Pseudomonas Aeruginosa, Staphylococcus Aureus |
| Patient 25 | Pseudomonas Aeruginosa | Pseudomonas Aeruginosa |
| Patient 26 | Pseudomonas Aeruginosa | Pseudomonas Aeruginosa |
| Patient 27 | Pseudomonas Aeruginosa, Achromobacter Xylosoxidans | Pseudomonas Aeruginosa, Achromobacter Xylosoxidans |
| Patient 28 | Pseudomonas Aeruginosa | Pseudomonas Aeruginosa |
| Patient 29 | Alcaligenes Xylosoxidans | Pseudomonas Aeruginosa, Achromobacter Xylosoxidans, Staphylococcus aureus, Stenotrophomonas Maltophilia, Alcaligenes Xylosoxidans |
| Patient 30 | Pseudomonas Aeruginosa, Stenotrophomonas Maltophilia | Pseudomonas Aeruginosa, Staphylococcus Aureus, Proteus Mirabilis |
| Patient 31 | Staphylococcus Aureus, Stenotrophomonas Maltophilia | Pseudomonas Aeruginosa |
| Patient 32 | Serratia Marcescens, Mycobacterium Abscessus | Serratia Marcescens, Klebsiella Pneumoniae |
| Patient 33 | Pseudomonas Aeruginosa, Proteus Mirabilis | Pseudomonas Aeruginosa, Proteus Mirabilis, Staphylococcus Aureus, Achromobacter Xylosoxidans, Moraxella Catarrhalis |
| Patient 34 | Pseudomonas Aeruginosa Staphylococcus Aureus | Staphylococcus Aureus, Haemophilus Influenzae |
| Patient 35 | Pseudomonas Aeruginosa, Pandorea Pulmonicola | Pseudomonas Aeruginosa, Pandorea Pulmonicola, Escherichia Coli |
| Patient 36 | Pseudomonas Aeruginosa, Staphylococcus Aureus | sterile |
| Patient 37 | Pseudomonas Aeruginosa, Staphylococcus Aureus, Alcaligenes Xylosoxidans, Acinetobacter Baumanii, Stenotrophomonas Maltophilia, Achromobacter xylosoxidans | sterile |
| Patient 38 | Pseudomonas Aeruginosa | sterile |
| Patient 39 | Pseudomonas Aeruginosa, Staphylococcus Aureus | sterile |
| Patient 40 | Pseudomonas Aeruginosa, Staphylococcus Aureus, Achromobacter Xylosoxidans | sterile |
