# Supplementary figures and images for: Impact of gram negative bacteria airway recolonization on the occurrence of chronic lung allograft dysfunction after lung transplantation in a population of cystic fibrosis patients
Source: BMC Microbiol. 2018 Aug 20;18:88. doi: 10.1186/s12866-018-1231-7 (PMC6102836; doi:10.1186/s12866-018-1231-7)

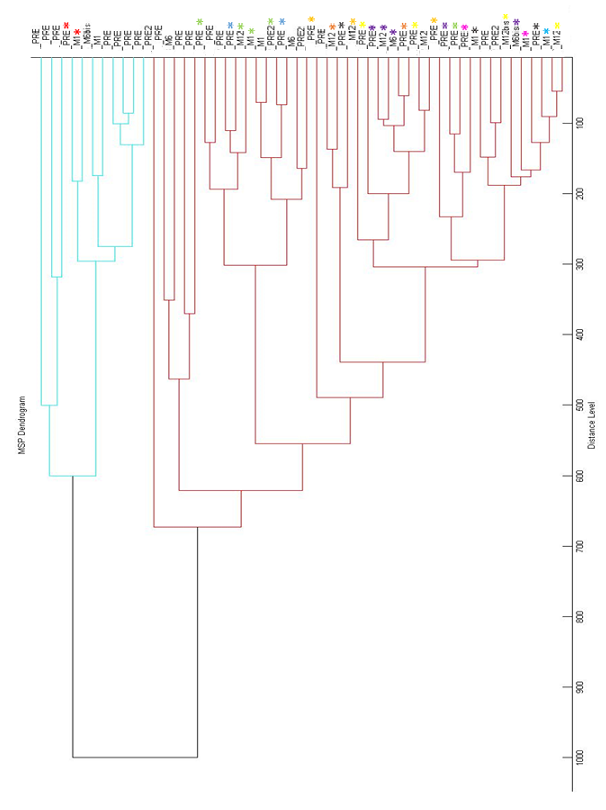

Supplement: Supplementary file 2 — Pseudomonas aeruginosa dendrogram. MSP dendrogram performed using Biotyper v 3.0, including 52 spectra of Pseudomonas aeruginosa isolated before, at one month, six months or twelve months after lung transplant for the 13 patients for which data were available. * indicates isolates of the same patient belonging to the same strain. Each color is specific for one patient. (DOCX 120 kb) [file 12866_2018_1231_MOESM2_ESM.docx]
