# Supplementary material for: Impact of gram negative bacteria airway recolonization on the occurrence of chronic lung allograft dysfunction after lung transplantation in a population of cystic fibrosis patients
Source: BMC Microbiol. 2018 Aug 20;18:88. doi: 10.1186/s12866-018-1231-7 (PMC6102836; doi:10.1186/s12866-018-1231-7)
Supplement: Supplementary file 3 — Best post-lung transplant Forced expiratory volume in 1 s (FEV1) in the three groups: GNB recolonization, GNB de-novo colonization, exempt of GNB. Best post-lung transplant Forced expiratory volume in 1 s (FEV1) in the three groups: GNB recolonization, GNB de-novo colonization, exempt of GNB expressed in percentage of the expected value. (DOCX 13 kb) [file 12866_2018_1231_MOESM3_ESM.docx]

| Recolonization FEV1 (%) | De-novo colonization FEV1 (%) | Exempt of GNB FEV1 (%) |
| --- | --- | --- |
| 95 | 108 |  |
| 72 | 91 | 91 |
| 87 |  | 62 |
| 101 | 80 | 76 |
| 142 | 89 | 100 |
| 106 | 75 |  |
| 64 | 102 |  |
| 69 |  |  |
| 62 |  |  |
| 71 |  |  |
| 78 |  |  |
| 102 |  |  |
| 76 |  |  |
| 94 |  |  |
| 99 |  |  |
| 55 |  |  |
| 61 |  |  |
| 88 |  |  |
| 76 |  |  |
| 82 |  |  |
| 96 |  |  |
| 83 |  |  |
| 84 |  |  |
| 78 |  |  |
| 69 |  |  |
| 95 |  |  |
| 81 |  |  |
| 43 |  |  |
